# Dysregulated calcium signaling in the aged primate association cortices: vulnerability to Alzheimer’s disease neuropathology

**DOI:** 10.3389/fnagi.2025.1610350

**Published:** 2025-07-15

**Authors:** Amy F. T. Arnsten, Isabella Perone, Min Wang, Shengtao Yang, Stacy Uchendu, Dinara Bolat, Dibyadeep Datta

**Affiliations:** ^1^Department of Neuroscience, Yale Medical School, New Haven, CT, United States; ^2^Department of Psychiatry, School of Medicine, Yale University, New Haven, CT, United States

**Keywords:** prefrontal cortex, entorhinal cortex, calpain-2, cAMP, primate, inflammation, pT217Tau

## Abstract

The common, late onset form of Alzheimer’s disease (AD) selectively impacts higher brain circuits, with tau pathology and neurodegeneration preferentially afflicting glutamatergic neurons in the limbic and association cortices. Understanding this selective vulnerability may help reveal the etiology of sporadic AD and therapeutic targets for prevention. The current review describes that these vulnerable circuits express magnified calcium signaling needed for higher cognition and memory, but that heightened calcium signaling becomes toxic when dysregulated by age and inflammation. Many of the earliest pathological events in AD are challenging to study in human brain, as proteins such as tau rapidly dephosphorylate postmortem. However, they can be studied in aging macaques, who are all APOE-ε4 homozygotes and naturally develop cognitive deficits, calcium dysregulation, synapse loss, tau and amyloid pathology and autophagic degeneration, including elevated plasma pT217Tau, a new blood biomarker of incipient AD. High resolution nanoscale imaging of aging macaque brains reveals the earliest stages of soluble tau pathology and its relationships with Aβ_42_ and calcium signaling. These data indicate that inflammation erodes regulation of calcium signaling leading to the activation of calpain-2, which drives tau hyperphosphorylation, APP cleavage to Aβ_42_ and autophagic degeneration. These in turn propel further calcium dysregulation to drive vicious cycles. Restoring calcium dysregulation, e.g., with calpain-2 inhibitors, thus may be a rational strategy for slowing or preventing AD pathology. Recent data show that an agent that reduces GCPII inflammation and restores mGluR3 regulation of calcium reduced tau pathology in aged macaques, encouraging this approach. Targeting inflammation and dysregulated calcium may be especially helpful for patients who are APOE-ε4 carriers and insufficiently aided by current anti-amyloid antibody treatments.

## Introduction

Dysregulated calcium (Ca^2+^) signaling has long been recognized as an etiological factor in Alzheimer’s disease (AD) pathology (Alzheimer's Association Calcium Hypothesis Workgroup, 2017; Camandola and Mattson, 2011; Guo et al., 1996; Khachaturian, 1991; Saito et al., 1993; Stutzmann, 2007; Webber et al., 2023). This review will discuss how high levels of Ca^2+^ are needed for neurons involved in higher cognition and memory formation, and how these same neurons are the target of AD pathology when Ca^2+^ becomes dysregulated by age and/or inflammation.

The review focuses on early etiological events, as these are most likely to be amenable to meaningful therapeutic interventions. Early changes often involve alterations in the phosphorylation state of proteins, e.g., hyperphosphorylation of tau as a key event leading to tau detaching from microtubules and the eventual formation of neurofibrillary tangles (NFTs). However, proteins such as tau rapidly dephosphorylate postmortem when they are still in an early, soluble state, and thus these important changes cannot be seen in human postmortem brains except in biopsy samples (Matsuo et al., 1994; Wang et al., 2015). In contrast, soluble phosphorylated tau can be seen in brains from aging macaques, where minimal postmortem intervals are possible, e.g., with perfusion fixation. The aging macaque is particularly useful for studying early changes relevant to sporadic AD, as macaques naturally develop neuroinflammation, synapse loss, amyloid and tau pathology, autophagic degeneration and cognitive deficits with advancing age. Macaques have well-developed association cortices, the focus of tau pathology in AD- and thus we are able to study why excitatory neurons in the limbic and association cortices with high levels of Ca^2+^ signaling are particularly vulnerable to pathology (Arnsten et al., 2021b). It is also noteworthy that macaques are APOE-ε4 homozygotes, which propels many aspects of neuroinflammation, Ca^2+^ dysregulation and AD pathology, helping to elucidate why this genotype increases risk of sporadic AD (Arnsten et al., 2021a; Arnsten et al., 2019; Datta and Arnsten, 2025). Thus, the aging macaque is useful for learning how inflammation contributes to Ca^2+^ dysregulation and the rise in AD pathologies, and how these molecular mechanisms relate to the evolutionary expansion of higher cognitive circuits, and the dementia that ensues with their neurodegeneration.

## Increased intracellular Ca^2+^ signaling across the cortical hierarchy: relationship to tau pathology in AD

**Figure 1:**
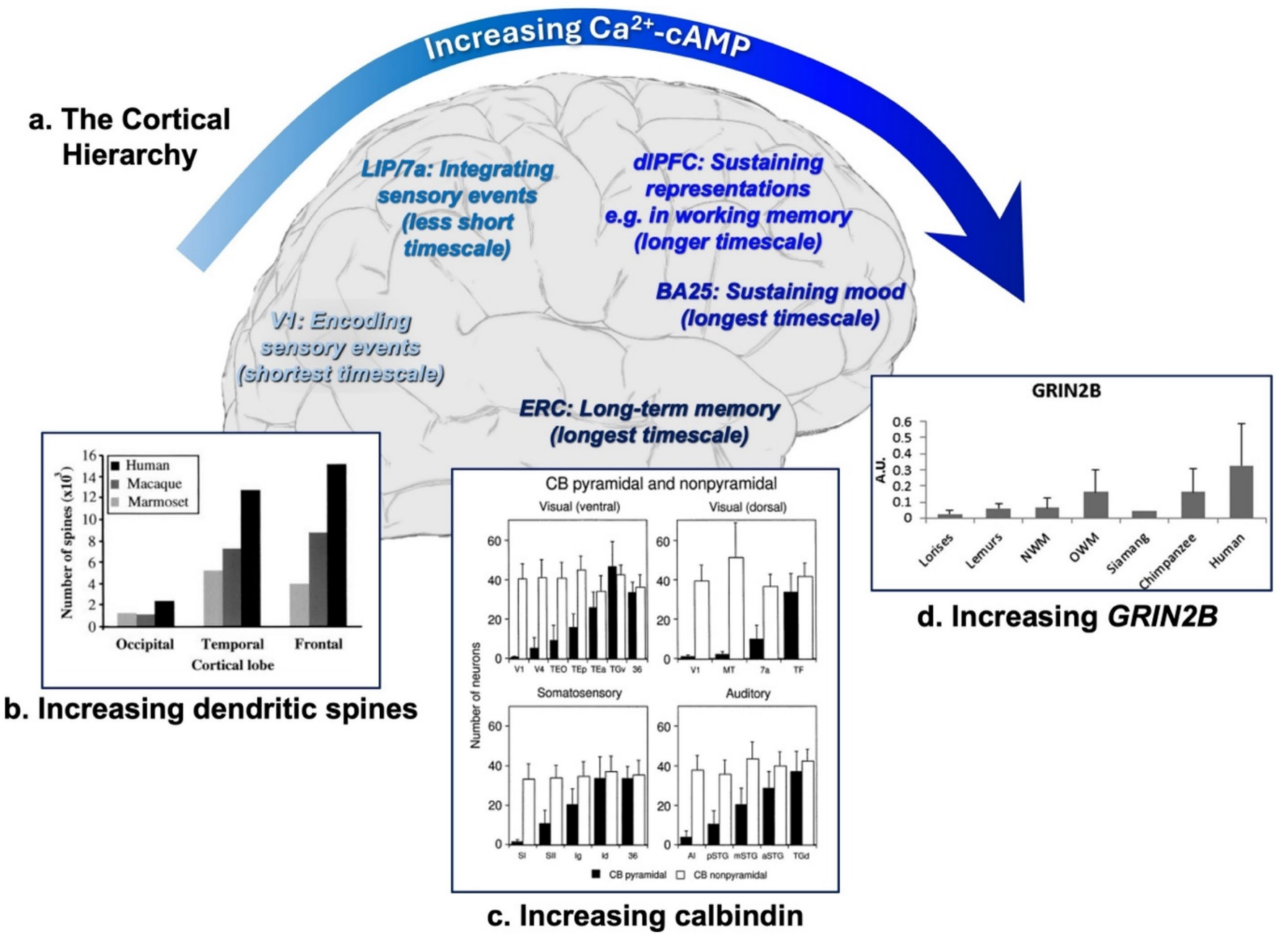
Increasing timescales, dendritic complexity and Ca^2+^-cAMP signaling across the cortical hierarchy in primates. **(a)** Schematic of the cortical hierarchy on a human brain, describing the increases in timescales from the briefest timescale in primary visual cortex area V1 to increasing longer timescales in association and limbic cortices (LIP/7a = parietal association cortices; dlPFC = dorsolateral prefrontal cortex; BA25 = Brodman area 25, the subgenual cingulate; ERC = entorhinal cortex). Timescale information is based on Murray et al. (2014). **(b)** The number of dendritic spines on a layer 3 pyramidal cell increases across the cortical hierarchy and across primate evolution. From Elston et al. (2001). **(c)** The expression of the Ca^2+^-binding protein, calbindin (*CALB1*), in macaque cortex increases across the cortical hierarchy in pyramidal cells, but not interneurons. From Kondo et al. (1999). **(d)** The expression of the NMDAR-GluN2B subunit, encoded by *GRIN2B*, that fluxes the highest levels of Ca^2+^, increases across primate evolution in the dlPFC. From Muntané et al. (2015). Both *GRIN2B* and *CALB1* expression increase across the human cortical hierarchy (Burt et al., 2018).

The cortex is highly organized in primates with a remarkable hierarchical, lattice-like configuration (Magrou et al., 2024). Thus, there are multiple differences as one proceeds from the primary sensory cortices, to the sensory association cortices, to higher cognitive association cortices, the limbic cortices mediating emotion, and finally to the generation of long-term memories by entorhinal cortex and hippocampus (Figure 1a). Computational analyses have found increasing timescales across this hierarchy, e.g., where the firing of a neuron at any one moment is increasingly influenced by its previous firing, as occurs with greater integration of information in sensory association cortex, prolonged representations of information in memory, and sustained mood states (Arnsten et al., 2021c; Murray et al., 2014). These functional differences across the cortical hierarchy correlate with increasing numbers of connections on dendritic spines (Elston et al., 2001; Elston et al., 2011) (Figure 1b), and increased expression of genes related to Ca^2+^ signaling, including the NMDA receptor subtype, GluN2B (*GRIN2B*) that closes slowly and fluxes the highest levels of Ca^2+^, and the Ca^2+^ binding protein, calbindin (*CALB1*), an indication of high Ca^2+^ use by a neuron (Burt et al., 2018). Studies of calbindin protein expression in macaques show that this hierarchical expression is due to increasing calbindin in pyramidal cells, not interneurons (Kondo et al., 1999) (Figure 1c), consistent with the increasing connections on pyramidal cell spines. Cytosolic Ca^2+^ levels are often increased by cAMP signaling (Arige and Yule, 2022) (and see below), and proteomic studies of human brain have also revealed a gradient in cAMP signaling across the cortical hierarchy, with greater expression of the phosphodiesterase *PDE4D* and the metabotropic receptor *GRM3* (mGluR3) in the dorsolateral prefrontal cortex (dlPFC) than in V1, both of which regulate cAMP-PKA signaling (Carlyle et al., 2017).

**Figure 2:**
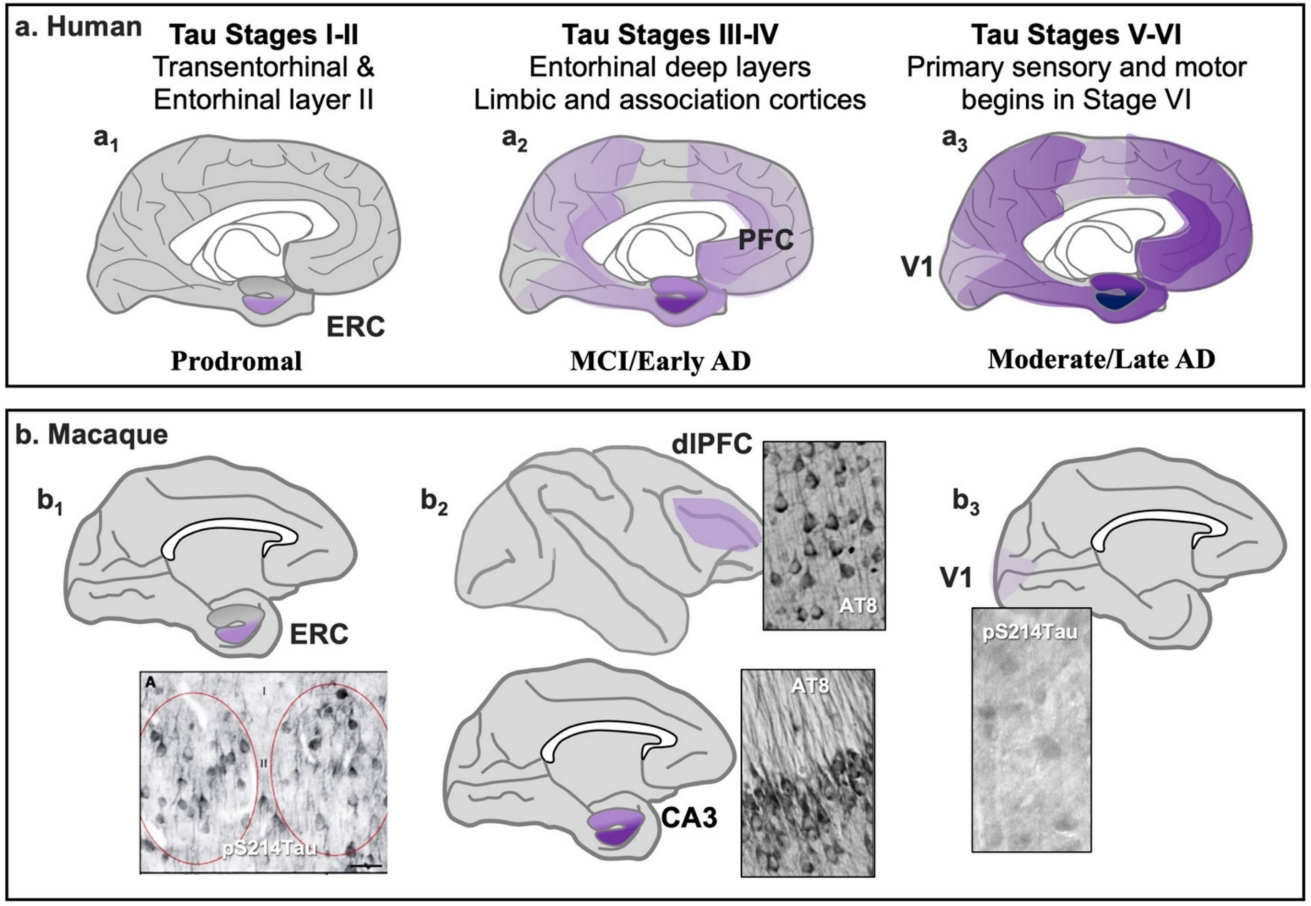
The progression of tau pathology in the cortex of patients with sporadic Alzheimer’s disease **(a)** and in aging macaques **(b)**. **(a)** Fibrillated tau pathology is first seen in cortex in trans-entorhinal and entorhinal cortices **(a**_**1**_**)**; it then progresses to interconnected hippocampal and limbic/association cortical circuits **(a**_**2**_**)**; and only begins to afflict the primary sensory and motor cortices at end stage disease **(a**_**3**_**)**. Based on Arnsten et al. (2021a). **(b)** A similar progression and pattern of soluble tau pathology is seen in aging macaques, with early stage tau pathology (pS214Tau) first seen in entorhinal cortex as young as middle age **(b**_**1**_**)** which becomes fibrillated at later ages. Tau pathology is next seen in hippocampus and association cortices **(b**_**2**_**)**, with the primary visual cortex unaffected even in the oldest animals **(b**_**3**_**)**. Based on Arnsten et al. (2021b) with figures from Paspalas et al. (2018) **(b**_**1**_**)**, Arnsten et al. (2021b) **(b**_**2**_**)**, and Carlyle et al. (2014) **(b**_**3**_**)**.

Intriguingly, there are also parallel differences across primate evolution, with expansion of the number of spines (Figure 1b) (Elston et al., 2001; Elston et al., 2011), and of *GRIN2B* expression (Figure 1d) (Muntané et al., 2015), from simple primates to human brains. These species differences are also highly relevant to AD etiology. For example, many neuroinflammatory mechanisms expand and/or change from mouse to human (Kodamullil et al., 2017), and the cortical hierarchy is much more subtle in mice (Gilman et al., 2017; Magrou et al., 2024). Thus, primate models can be particularly useful for understanding early etiological factors that may not be present in mouse cortex and that especially afflict excitatory neurons at the higher levels of the cortical hierarchy. In this regard it is noteworthy that the pattern of calbindin expression in pyramidal cells across the cortical hierarchy fits remarkably well with the pattern of neurons that develop tau pathology and degenerate in AD (with a few remarkable exceptions described below) (Figure 2a).

In patients with sporadic AD, tau pathology in cortex begins in layer II of the transentorhinal and entorhinal cortices (ERC; Tau stages I-II), and then spreads to closely interconnected circuits in the limbic/association cortices and hippocampus (Tau stages III-IV) (Braak et al., 2011; Hyman et al., 1984). The layer II cell islands of the entorhinal cortices are a key site for funneling inputs from most of the association cortices into the hippocampus for the formation of new memories (Hyman et al., 1984), and thus are a key anatomical hub for recent memory, and also for the seeding of tau pathology through higher cognitive and memory circuits (Kaufman et al., 2018). For example, at later stages tau pathology can be seen in the dlPFC that subserves abstract reasoning, working memory and the executive functions and correlates with cognitive deficits (Giannakopoulos et al., 2003). At Tau stage V, pathology can be seen throughout the association cortices, but it only reaches the primary visual and auditory cortices at end stage disease (Tau stage VI) (Braak et al., 2011; Lewis et al., 1987). This pattern generally fits with the progression of symptoms, from recent memory deficits to a range of cognitive deficits (i.e., dementia), including the progressive loss of long-term memories, with sensory-motor experience preserved until the latest stages. As we will see below, there is a similar pattern and progression of early stage, soluble tau pathology in the aging macaque cortex (Figure 2b). Recent studies in humans also indicate that tau is becoming phosphorylated earlier than expected, as plasma levels of tau phosphorylated at threonine 217 (pT217Tau) is an emerging, early biomarker that heralds future AD (Barthélemy et al., 2024; Mendes et al., 2024; Palmqvist et al., 2020; Pandey et al., 2025).

Calbindin expression is decreased in AD brains (Lally et al., 1997), suggesting that the loss of its protective actions may contribute to AD pathology. The relationship between calbindin and tau pathology was directly studied in the dlPFC of patients with AD, where it was found that the layer III pyramidal cells in the dlPFC that express calbindin when younger and healthy are especially vulnerable to tau pathology and degeneration, while interneurons retained calbindin expression and did not degenerate (Hof and Morrison, 1991). Importantly, in both animals and humans, calbindin expression is lost with age (Datta et al., 2021; Erraji-Benchekroun et al., 2005), stress (Li et al., 2017) and/or inflammation (Reiken et al., 2022), and these are all risk factors for sporadic AD. There are also age-related reductions in other factors that normally regulate cAMP-PKA increases in Ca^2+^ signaling, including loss of PDE4A (Carlyle et al., 2014), PDE4D (Datta et al., 2021), mGluR3, and α2A-AR (Erraji-Benchekroun et al., 2005). As calbindin expression is an indication of high Ca^2+^ use by a cell, exploring why this subset of pyramidal cells express calbindin, and how it relates to their function, may provide clues to this selective neuronal vulnerability, and strategies for treatment.

Another important clue arises from the observation that tau pathology within neurons is first seen in distal dendrites (“neuropil threads” in postmortem human brain tissue) and then moves proximally into the soma, with the axon only afflicted last (Braak and Del Tredici, 2018). What is happening in distal dendrites with advancing age that initiates tau hyperphosphorylation? We have been probing this question by examining the distal dendrites and spines in macaque ERC and dlPFC at high resolution with immunoelectron microscopy (immunoEM) where we see evidence of increased intracellular Ca^2+^ signaling near glutamate synapses on spines, necessary to cognitive functioning, and the emergence of hyperphosphorylated tau when Ca^2+^ signaling becomes dysregulated with advancing age.

## Increased intracellular Ca^2+^ is needed for higher cognition and memory

**Figure 3:**
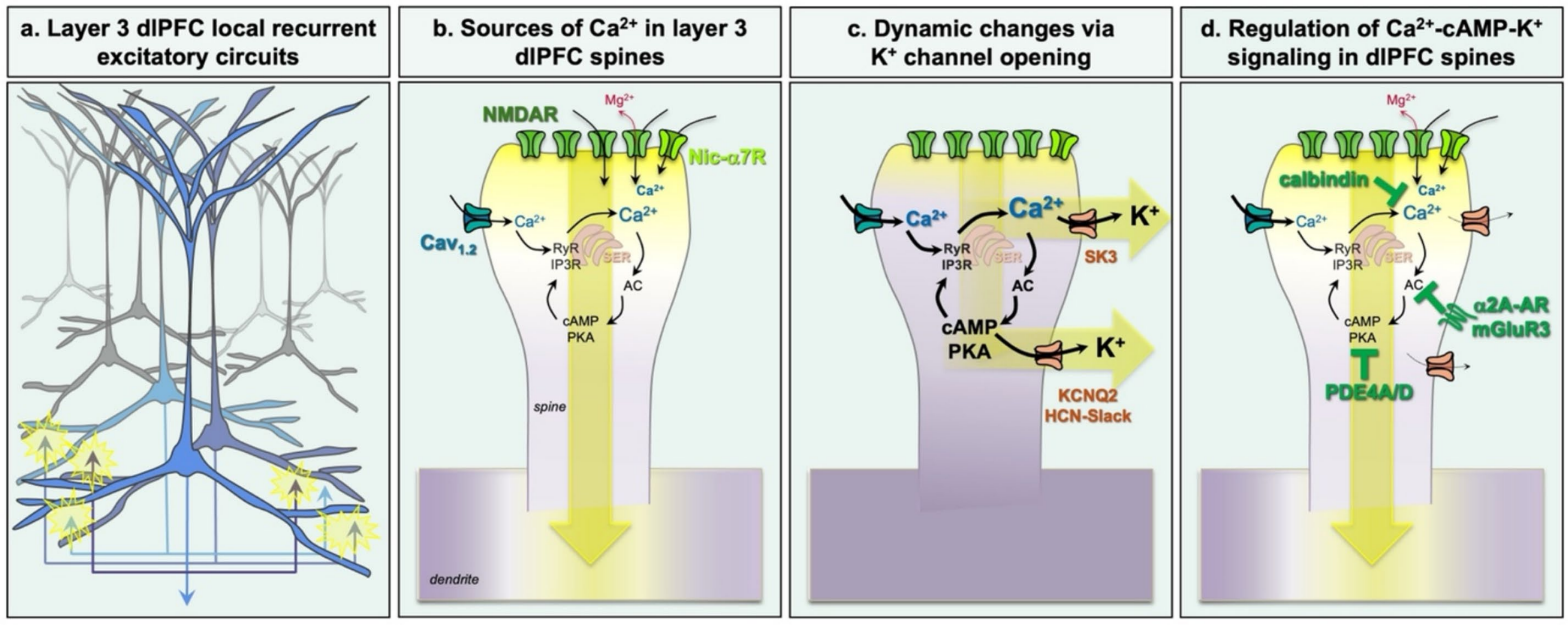
The layer III dlPFC pyramidal cell circuits that underlie higher cognition express increased Ca^2+^ signaling needed for higher cognition. **(a)** Extensive, local recurrent excitation is thought to subserve the sustained neuronal firing needed to represent information in working memory. **(b)** NMDAR neurotransmission on layer III dlPFC spines requires increased Ca^2+^ signaling which arises from multiple sources, including feedforward cAMP-PKA drive on internal Ca^2+^ release. **(c)** Layer III dlPFC dendritic spines also express high levels of potassium channels that are opened by calcium or cAMP-PKA signaling. Opening of these channels weakens synaptic efficacy and rapidly reduces neuronal firing. **(d)** Under healthy conditions, feedforward cAMP-calcium signaling is tightly regulated by the phosphodiesterases PDE4A and PDE4D, that catabolize cAMP, and by calbindin, which binds calbindin in the cytosol. The production of cAMP is also inhibited by mGluR3 and by α2A-AR, both of which reside on spines in primate dlPFC and enhance dlPFC neuronal firing by regulating cAMP- Ca^2+^-K^+^ channel signaling. See text for details.

The neural bases of working memory in the primate dlPFC has been studied for decades (Fuster and Alexander, 1971; Goldman-Rakic, 1995), and so there is a strong framework for examining the molecular regulation of these circuits and how they change with age. The persistent neuronal firing that keeps information “in mind” during working memory relies on the extensive, local recurrent excitatory synapses on dendritic spines, especially in layer III dlPFC (Goldman-Rakic, 1995) (Figure 3a). These neurons that are capable of representing information in working memory are called “Delay cells,” as they are able to sustain spatially-tuned firing for the memory of spatial location across the delay period in a spatial working memory task (Funahashi et al., 1989). More recently, we have learned that relatively high levels of intracellular Ca^2+^ signaling near the postsynaptic density (PSD) are needed to sustain this persistent firing (Arnsten et al., 2021c; Datta et al., 2024b). Indeed, layer III dlPFC pyramidal cells express an enriched constellation of Ca^2+^-related genes (e.g., *CALB1, GRIN2B, CACNA1C, KCNN3*), and intracellular Ca^2+^ is needed to sustain Delay cell firing during working memory (Datta et al., 2024b). Elevated intracellular Ca^2+^ levels near the PSD in their dendritic spines may come from a variety of sources, as schematized in Figure 3b:

- NMDA receptors, including those with GluN2B subunits, are essential to Delay cell firing (Wang et al., 2013). NMDAR-GluN2B close slowly and flux high levels of Ca^2+^ into the spine. In dlPFC they are found mostly in the PSD, but in other circuits, e.g., the subgenual cingulate, they are mostly at extrasynaptic locations where they may be a source of excessive calcium entry (Joyce et al., 2025a; Wang et al., 2013). These extrasynaptic NMDAR-GluN2B increase their expression in AD and may be especially important for the toxic effects of Ca^2+^ (Escamilla et al., 2024).
- Nic-α7R reside within glutamatergic synapses in layer III dlPFC and play a key, permissive role for NMDAR neurotransmission, a role normally performed by AMPAR (Yang et al., 2013). Nic-α7R flux both sodium and Ca^2+^ into the neuron, and may depolarize the PSD to sustain NMDAR actions.
- Internal Ca^2+^ release from the smooth endoplasmic reticulum (SER) near the PSD and throughout the dendritic spine, called the “spine apparatus” as the SER elaborates greatly in spines, and is a frequent feature of dlPFC spines (Arnsten et al., 2021c; Datta et al., 2024b). Ca^2+^ is released into the cytosol through both ryanodine (RyR) and IP3 receptors (IP3R).
- Voltage-gated Ca^2+^ channels also flux Ca^2+^ into the spine, and recent data show that Cav_1.2_ Ca^2+^ channels are focused on layer III dlPFC spines (Datta et al., 2024b). These channels are often near the SER spine apparatus, where they may increase Ca^2+^-mediated Ca^2+^ release into the cytosol (Datta et al., 2024b), similar to Cav_1.2_ actions in the heart (Dixon, 2022).

It is well-established that cAMP-PKA and Ca^2+^ signaling interact extensively, where cAMP-PKA signaling increases Ca^2+^ entry into the cytosol, and Ca^2+^ in turn can increase the production of cAMP (see Arige and Yule, 2022 for excellent review). A variety of evidence suggests that these feedforward interactions are prominent within the dendritic spines of layer III dlPFC pyramidal cells in primates. For example, a concentration of cAMP-related proteins can be seen on or near the SER spine apparatus (Arnsten et al., 2021c; Datta et al., 2024b). Cytosolic Ca^2+^ can in turn activate adenylyl cyclase 1 (AC1) to increase cAMP production, thus creating feedforward signaling (Figure 3b). Layer III dlPFC pyramidal cells are also enriched in cAMP-related transcripts (Arnsten and Datta, 2024), including AC1 (*ADCY1*), and the PKA anchoring protein AKAP5 (also called AKAP150) which anchors PKA, Cav_1.2_ and β-adrenoceptors as a signaling unit (Davare et al., 2001; Hall et al., 2007). It is noteworthy that this signature of feedforward cAMP- Ca^2+^ signaling does not seem to appear in layer III spines in the primary visual cortex, consistent with a hierarchical expression pattern across cortex (Arnsten et al., 2021c; Yang et al., 2018).

## High levels of intracellular Ca^2+^-cAMP signaling in dlPFC weaken connectivity

Although cAMP- Ca^2+^ signaling is needed to sustain dlPFC Delay cell firing during working memory, high levels of cAMP- Ca^2+^ signaling, e.g., during stress exposure, open K^+^ channels on spines that weaken effective connectivity and reduce firing (Figure 3c). These K^+^ channels include SK channels that are opened by Ca^2+^ (Datta et al., 2024b), KCNQ2 channels opened by PKA signaling (Galvin et al., 2020), and HCN-Slack channels that appear to form a complex opened by cAMP signaling in spines (Paspalas et al., 2013; Wang et al., 2007b; Wu et al., 2023). The levels of SK channel expression in particular may determine whether a neuron exhibits a *hypo-* or *hyper-*excitability response to high levels of Ca^2+^, where pyramidal cells like those in layer III dlPFC with high levels of SK3 channels *reduce* firing under conditions of very high intracellular Ca^2+^. A similar subset of pyramidal cells has been found in mouse medial prefrontal cortex, which have higher levels of IP3-mediated internal Ca^2+^ release that reduce neuronal activity via SK channel opening (Stutzmann et al., 2003). This rapid opening or closing of K^+^ channels allows the prompt coordination of cognitive state with arousal state, e.g., taking the energy-intensive dlPFC “off-line” during fatigue or sickness (see below), or swiftly switching control of behavior to more primitive circuits during danger (Arnsten et al., 2012). This rapid alteration in synaptic efficacy is termed Dynamic Network Connectivity, where high levels of Ca^2+^-cAMP-K^+^ signaling confer a “signature of flexibility” (Arnsten et al., 2012; Datta et al., 2023). These molecular actions are especially driven during conditions of uncontrollable stress through multiple mechanisms (Datta et al., 2024b; Joyce et al., 2025b), switching the control of behavior from more recently evolved, reflective circuits, to more primitive, reflexive circuits under conditions of threat (Arnsten, 2009). This can be seen in animals (Murphy et al., 1996), and humans (Qin et al., 2009), especially in response to an uncontrollable stressor (Baratta et al., 2023; Wanke and Schwabe, 2020). This may have survival value under some circumstances, e.g., in battle, but is counterproductive when higher cognitive processes are needed to deal with complex challenges.

With chronic stress there is actual loss of spines and dendrites that correlates with cognitive deficits (Hains et al., 2009; Liston et al., 2009; Liston et al., 2006; Radley et al., 2006; Woo et al., 2021). This can be seen in humans as well, where loss of prefrontal gray matter correlates with the number of aversive or traumatic events (Ansell et al., 2012).

Under healthy conditions the stress response is tightly regulated, as summarized in Figure 3d. In macaque, layer III dlPFC pyramidal cells express the Ca^2+^-binding protein, calbindin (Datta et al., 2021), the phosphodiesterases PDE4A/D which are anchored to the SER spine apparatus to regulate feedforward cAMP- Ca^2+^ signaling and reduce Ca^2+^ release from the SER (Carlyle et al., 2014; Datta et al., 2020a; Datta et al., 2021), and receptors on the spine membrane that inhibit the production of cAMP: α2A-AR (Wang et al., 2007b) and mGluR3 (Jin et al., 2018). For example, stimulation of α2A-AR (Wang et al., 2007b) or mGluR3 (Jin et al., 2018) enhances Delay cell firing, and chronic α2A-AR stimulation with guanfacine protects pyramidal cells from spine loss under conditions of chronic stress or hypoxia in rodent models (Hains et al., 2015; Kauser et al., 2013; Kauser et al., 2016). mGluR3 are not only stimulated by glutamate, but by NAAG, which is co-released with glutamate and is selective for mGluR3 (Yang et al., 2022). This may render mGluR3 regulation particularly vulnerable to inflammation, when GCPII inflammatory signaling catabolizes NAAG and markedly reduces dlPFC Delay cell firing (Jin et al., 2018; Yang et al., 2022).

## Loss of regulation with age and/or inflammation

**Figure 4:**
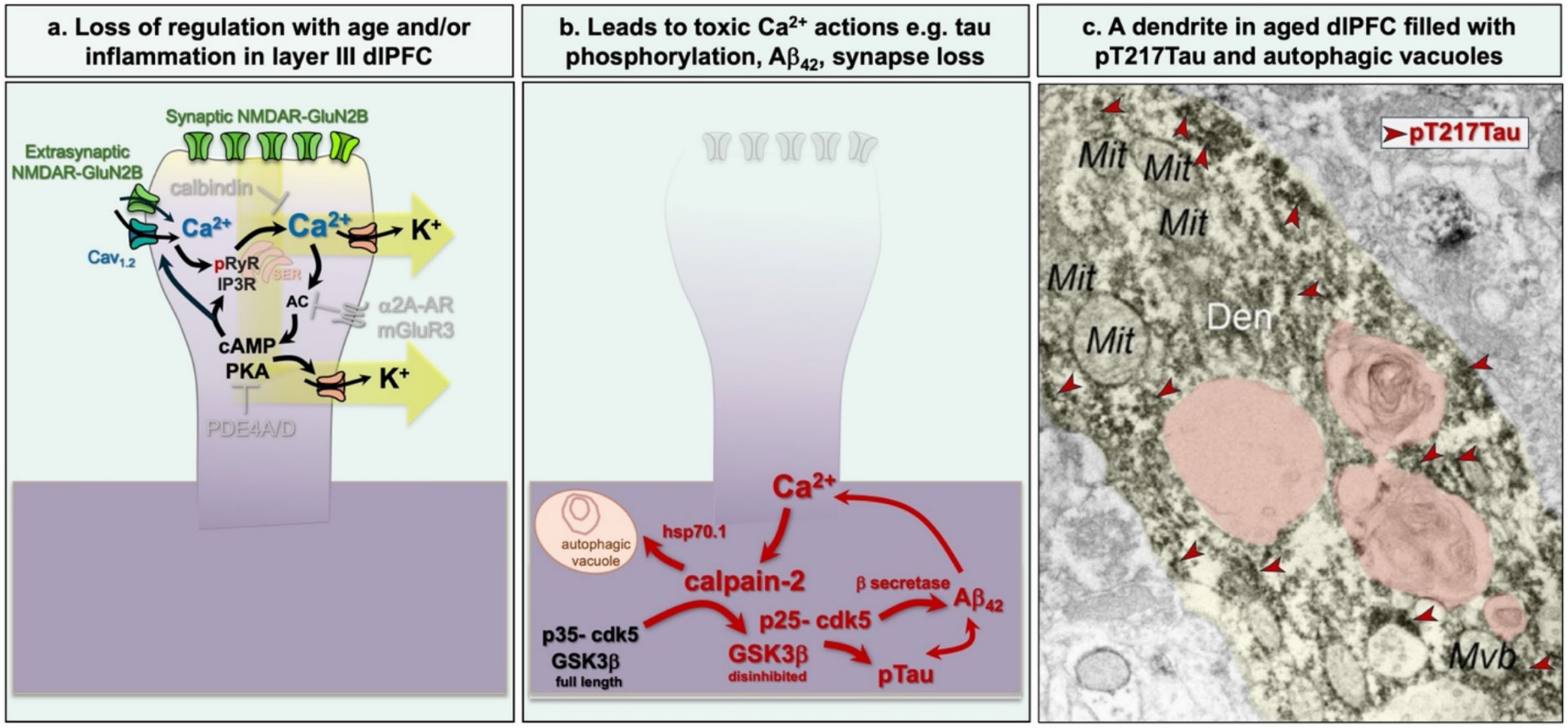
Loss of cAMP- Ca^2+^ regulation with age and/or inflammation leads to loss of firing and AD pathology. **(a)** Schematic diagram showing that loss of calbindin, PDE4s, and α2A-AR/mGluR3 regulation during aging/inflammation dysregulates cAMP- Ca^2+^ signaling, opening K^+^ channels and reducing dlPFC neuronal firing. Extrasynaptic NMDAR-GluN2B in aged cortex may also contribute to excessive cytosolic Ca^2+^. **(b)** When cytosolic Ca^2+^ levels are high enough to activate calpain-2, multiple toxic actions occur, including calpain-2 cleavage and disinhibition of GSK3β and p35-cdk5 to p25-cdk5, which hyperphosphorylated tau, and cleavage and activate of heatshock protein 70.1 (hsp70.1) to induce lysosomal abnormalities and autophagic degeneration. Activation of p25-cdk5 also increases β-secretase cleavage of APP to Aβ_42_. **(c)** An example of a dendrite from a layer III pyramidal cell in the dlPFC of an aged macaque with extensive pT217Tau aggregated on microtubules (a subset indicated by red arrowheads), and multiple autophagic vacuoles (orange pseudocoloring) showing early stages of neurodegeneration. From Datta et al. (2024a). Den, dendrite; mit, mitochondrion; Mvb, multivesicular body.

Under healthy conditions, feedforward Ca^2+^-cAMP-PKA-K^+^ channel signaling in layer III dlPFC is tightly regulated by calbindin, PDE4s, mGluR3s and α2A-ARs (Figure 3d). However, these regulatory mechanisms are lost with age and/or inflammation, leading to extensive K^+^ channel opening and a variety of toxic events including hyperphosphorylation of tau (Figure 4) and the rise in complement inflammation (Datta et al., 2020b). Interestingly, calbindin remains in aged dlPFC interneurons but is lost from layer III pyramidal cells (Datta et al., 2021), which may help to explain the greater vulnerability of pyramidal cells to tau pathology. Age and/or inflammation can also unanchor and reduce the expression of the PDE4s (Carlyle et al., 2014; Datta et al., 2021), and inflammation increases the expression of GCPII which catabolizes NAAG, reducing mGluR3 regulation of cAMP-PKA signaling (Yang et al., 2022). Similar decreases in message for calbindin, α2A-AR and mGluR3 signaling, and increases in complement can be found in the aged human dlPFC (Erraji-Benchekroun et al., 2005). Dysregulated cAMP-PKA signaling can in turn further increase Ca^2+^ entry through calcium channels and NMDAR into neurons (Hall et al., 2007; Skeberdis et al., 2006). High levels of PKA activity also increase Ca^2+^ leak from the SER into the cytosol by phosphorylating type II ryanodine receptors, displacing calstabin-2, also known as FKBP12.6, which normally prevents this leakage (Lacampagne et al., 2017). Other post-translational modifications such as oxidation or nitrosylation of RyR2, can also disassociate RyR2 from calstabin-2 (Shan et al., 2010; Wehrens et al., 2005; Wehrens et al., 2006). PKA-phosphorylated ryanodine receptors (pS2808RyR2) can be seen in the aged macaque dlPFC where their levels correlate with the rise in PKA phosphorylated tau (Arnsten et al., 2021b). Importantly, pS2808RyR2 is also evident in AD brain (Lacampagne et al., 2017), as well as in the brains of patients who died from COVID-19, who also show reduced PDE4 and increased GCPII, PKA signaling and hyperphosphorylation of tau (Reiken et al., 2022). These data emphasize the close relationships between aging, inflammation, and AD pathology.

Ca^2+^ leak from the SER is noteworthy in that it can be seen in multiple models and disease conditions. It is seen with advanced age in the rodent ERC (Gant et al., 2018), and at young ages in mouse AD models (Chakroborty et al., 2009). It is noteworthy that the PS1 and PS2 mutations that cause autosomal dominant AD also cause massive Ca^2+^ leak from the SER, although through more direct disruptions (Chami and Checler, 2020; Guo et al., 1996; Tu et al., 2006). Thus, stabilizing internal Ca^2+^ release has been suggested as an important therapeutic approach (Chakroborty et al., 2012).

**Figure 5:**
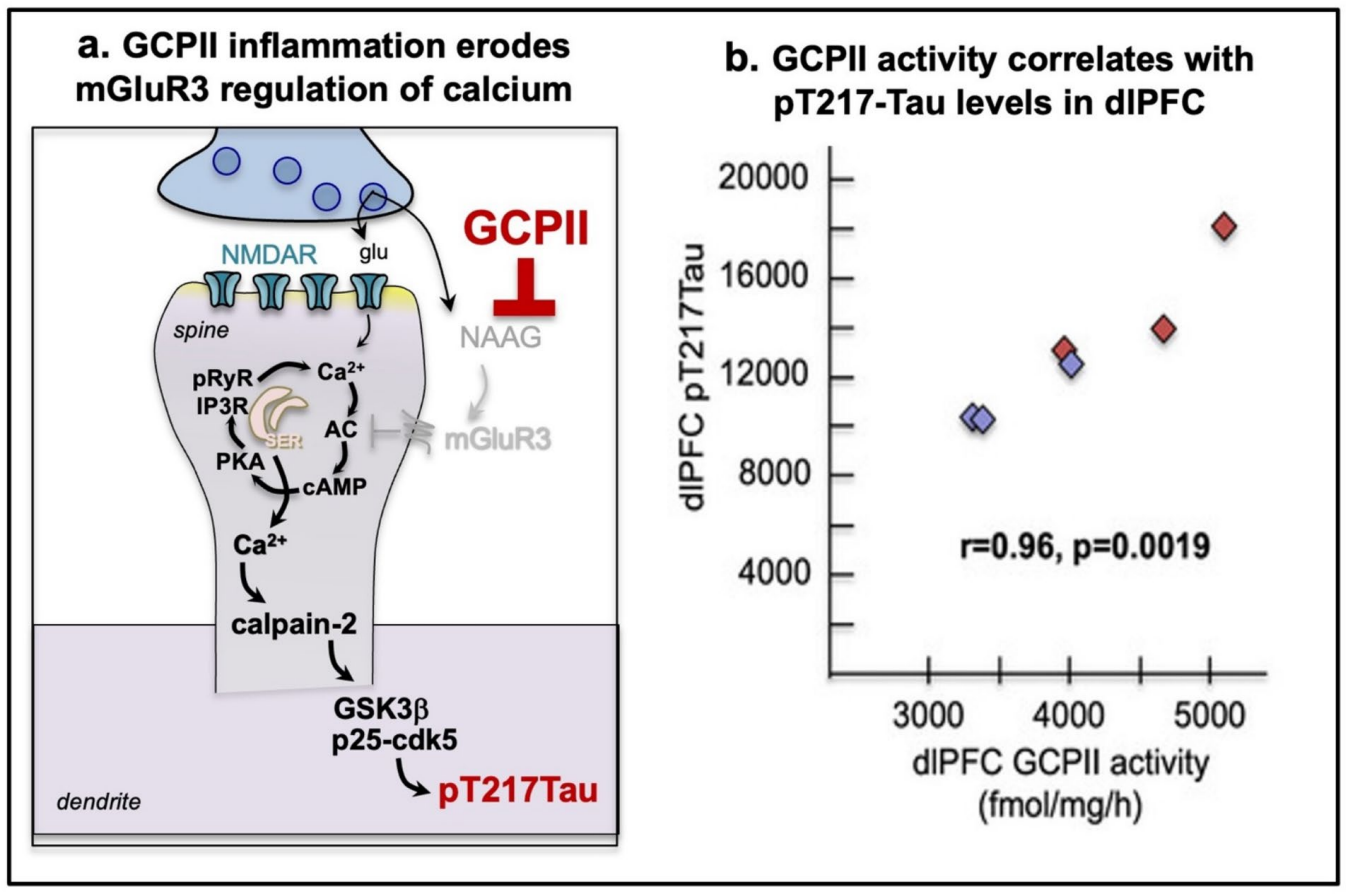
GCPII (glutamate carboxypeptidase II) inflammation has detrimental actions in the aged primate dlPFC. **(a)** A schematic diagram showing GCPII inflammation leads to elevated pTau. GCPII catabolizes NAAG, the endogenous ligand for mGluR3 that is co-released with glutamate and is selective for mGluR3. Thus, GCPII erodes mGluR3 regulation of feedforward cAMP-calcium signaling in primate dlPFC leading to toxic levels of Ca^2+^ in the cytosol. High levels of cytosolic Ca^2+^ activate calpain-2, which cleaves and activates GSK3β and p25-cdk5 of hyperphosphorylated tau, e.g., at pT217Tau. **(b)** The levels of GCPII activity in the aged macaque dlPFC highly correlate with levels of pT217Tau. From Bathla et al. (2023).

In contrast to Ca^2+^ leak, the role of GCPII inflammation has not received much attention, but it may be especially relevant to the role of inflammation in driving sporadic AD pathology (Figure 5a). GCPII activity in aged macaque brain highly correlates with pT217Tau levels (Figure 5b) (Bathla et al., 2023), and has large, detrimental effects on dlPFC neuronal firing (Jin et al., 2018; Yang et al., 2022). Thus, this mechanism may be particularly powerful in primate brain where mGluR3s have a new and expanded, post-synaptic protective role compared to rodents (Jin et al., 2018).

Very high levels of Ca^2+^ in the cytosol can activate calpain-2 which then cleaves and activates other destructive pathways (Figures 4b,5a; reviewed in Arnsten and Baudry, 2023; Datta and Arnsten, 2025). This appears to be an important event in human AD brains, as activated calpain-2 is seen in association with neurofibrillary tangles (Adamec et al., 2002; Grynspan et al., 1997; Nixon, 2003), and upregulation of calpain activity heralds tau pathology (Kurbatskaya et al., 2016) and correlates with cognitive deficits (Ahmad et al., 2018). There are multiple mechanisms by which activation of calpain-2 can increase AD pathology (Figure 4b). For example, calpain-2 directly cleaves and disinhibits GSK3β (Goñi-Oliver et al., 2007), and cleaves and p35 to p25 to activate both cdk5 (Maitra and Vincent, 2022) and GSK3β (Chow et al., 2014), two of the major kinases that hyperphosphorylates tau. p25-cdk5 activation additionally activates β-secretase and the cleavage of APP to Aβ_42_ (Wen et al., 2008). Ca^2+^ also increases multiple mechanisms involved with actin reorganization (Briz and Baudry, 2017; Mikhaylova et al., 2020), which can lead to protein kinase C-mediated spine loss (Calabrese and Halpain, 2005). Calpain-2 cleavage of hsp_70.1_ drives autophagic degeneration (Sahara and Yamashima, 2010) and weakens lysosomal function (Yamashima et al., 2024), and one can see extensive autophagic degeneration of aged dlPFC dendrites that are filled with pT217Tau (Figure 4c; Datta et al., 2024a). Thus, high levels of Ca^2+^ can promote all the major pathological indices of AD. As described below, pTau and Aβ_42_ further increase Ca^2+^ dysregulation, thus increasing vicious cycles that cause loss of function and ultimately neuronal degeneration.

## A similar molecular signature is seen in layer II of entorhinal cortex

**Figure 6:**
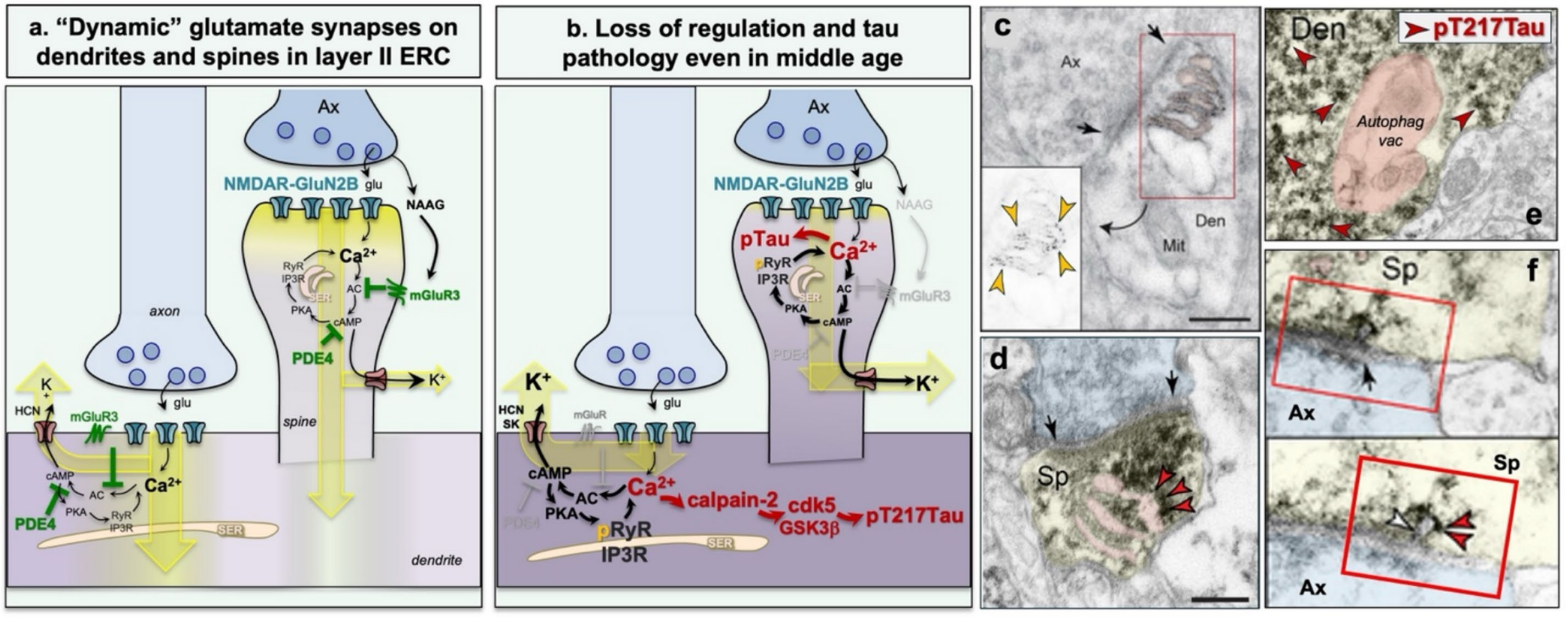
Excitatory neurons in layer II of the entorhinal cortex, the most vulnerable cortical neurons in AD, show molecular regulation similar to layer III dlPFC. **(a)** Schematic illustration showing molecular regulation with increased intracellular Ca^2+^ signaling similar to dlPFC (see Figure 4), with two important exceptions that likely make these neurons more vulnerable: a large percentage of excitatory synapses on dendrites where there is extensive smooth endoplasmic reticulum (SER) containing Ca^2+^, and the absence of calbindin, even in the young, healthy ERC. **(b)** Schematic illustration of the aging ERC with Ca^2+^ dysregulation and tau pathology (see Figure 4B for details). **(c)** Example of PKA phosphorylated ryanodine receptors (pRyR2) on the SER in an ERC dendrite, which causes Ca^2+^ leak into the cytosol. From Paspalas et al. (2018). **(d)** Example of pT217Tau on the SER in an ERC dendritic spine. From Datta et al. (2024a). **(e)** Example of autophagic degeneration in an ERC dendrite filled with pT217Tau (a few examples highlit with red arrowheads); the autophagic vacuole is pseudocolored orange. Den = dendrite. From Datta et al. (2024a). **(f)** Examples of pT217Tau (red arrowheads) “seeding” between neurons in the macaque ERC. The spine is pseudocolored in yellow; the axon terminal in blue; the seeding appears to occur at the synapse. From Datta et al. (2024a).

The layer II cell islands of the ERC are the first to show tau pathology in cortex, as early as middle age. We have been studying these cells in macaque to try to learn why they may be more vulnerable than other cortical neurons. A recent immunoEM analysis shows that layer II of the macaque entorhinal cortex expresses a similar pattern of “flexibility/vulnerability” as layer III of the dlPFC (Figure 6a) (Datta et al., 2023). This may relate to the similar roles of the dlPFC and the ERC in generating representations that are influenced by environmental conditions/arousal state, e.g., with HCN channel opening narrowing memory fields/grid scales (Giocomo et al., 2011; Wang et al., 2007b). Layer II of the ERC appears to have many of the same regulatory mechanisms as layer III dlPFC, e.g., PDE4D concentrated on the SER and postsynaptic mGluR3 (Figure 6a) (Datta et al., 2023). However, a major exception is that the layer II ERC cell islands most vulnerable to tau pathology never express calbindin in either human or macaque ERC (Beall and Lewis, 1992). Thus, these ERC cell islands may exhibit tau pathology starting in middle age due to their having a signature of increased Ca^2+^ signaling without the protection of calbindin expression even at young ages. Layer II of the ERC also is unique in having large numbers of glutamatergic synapses directly on the dendritic shafts of excitatory neurons in layer II (Domínguez-Álvaro et al., 2021) (schematically shown in Figures 6a,b), which may further increase vulnerability to degeneration given the extensive SER in dendrites.

Evidence of Ca^2+^dysregulation can be seen in the macaque ERC even in young middle age. For example, immunoEM reveals a remarkable elaboration of the SER under glutamate synapses on the dendrites of layer II ERC excitatory neurons with evidence of Ca^2+^ leak from its ryanodine receptors (p2808RyR2) (Paspalas et al., 2018) (Figure 6c). These middle-aged layer II ERC cell islands also already express pS214Tau (Paspalas et al., 2018). Biochemical analyses over a wider age range show further age-related decreases in PDE4D, and age-related increases in calpain-2, GCPII and pTau (pT181Tau, pT217Tau) in the macaque ERC, where GCPII inflammation reduces mGluR3 regulation of intracellular Ca^2+^ signaling (Bathla et al., 2025). pT217Tau can be seen accumulating on microtubules and over the SER in layer II dendrites even at early stages of the aging process (Figure 6d; Datta et al., 2024a). Thus, the ERC layer II cell islands are an early site of Ca^2+^ dysregulation and tau phosphorylation.

Ca^2+^ dysregulation can have a number of actions that increase AD pathology, and AD pathology can in turn drive Ca^2+^ dysregulation. The following sections will describe some of these actions in turn, including the complex, functional consequences of excessive Ca^2+^ on neuronal firing.

## The role of intracellular Ca^2+^ dysregulation in hyper- or hypo-excitability

The AD field is currently debating whether there is hyper- or hypo-excitability in early AD. The FDG-PET shows evidence of pervasive *hypo*activity of cortical circuits (Caselli et al., 2008; Huang et al., 2024), but as discussed below, multiple other perspectives, and especially those from mouse AD models, propose early *hyper*activity. Given Ca^2+^‘s effects on neuronal excitability, this issue is reviewed in this section.

The classic view of Ca^2+^ actions is that increased Ca^2+^ induces *hyper*excitability, consistent with it being a positively charged ion. For example, manipulations that increase Ca^2+^ release from the SER in mouse hippocampal neurons abnormally increase neuronal firing and impair memory (Yao et al., 2022). This is often coupled with Ca^2+^-induced increases in mitochondrial energy production (Denton, 2009), coordinating neuronal firing with energy demands (Rossi et al., 2019). The classic view also denotes that very high cytosolic Ca^2+^ levels are toxic, e.g., under conditions of stroke, when rapid increases in intracellular Ca^2+^ lead to Ca^2+^ overload of mitochondria and apoptosis, i.e., cell death (Rossi et al., 2019). Although it is sometimes presumed that this occurs in AD as well, neurons in AD actually die by autophagic degeneration, a slow process where the neuron gradually eats itself from within, and not by apoptosis (Okamoto et al., 1991; Yamashima, 2013). The Ca^2+^ dysregulation that occurs with aging, inflammation and AD is more subtle and more complex than in stroke, where toxic Ca^2+^ actions build slowly and are sustained over time (Yamashima, 2013). As described in the following section, under these conditions, many neurons in higher cortical circuits may actually show *reduced* neuronal firing due to opening of SK potassium channels (see below), as well as evidence of Ca^2+^ overload of mitochondria in the absence of apoptosis, complicating simple interpretations. As many recent studies of mouse AD models and human proteomic/transcriptomic data have assumed that neurons exhibit hyperexcitability in AD, this issue is discussed here in some detail.

Many of the higher cortical neurons that are the target of tau pathology in AD express high levels of SK potassium channels that are opened by Ca^2+^, and thus very high levels of cytosolic Ca^2+^ actually *reduce* rather than increase neuronal firing (Datta et al., 2024b). SK potassium channels are opened by Ca^2+^ and play a key role in reducing firing under conditions of high intracellular Ca^2+^ in some neurons (Sahu and Turner, 2021). For example, SK3 potassium channel expression is especially high in the layer III pyramidal cells in the human and macaque dlPFC that are especially vulnerable to tau pathology (Datta et al., 2024b), and their expression increases across the cortical hierarchy in humans in correspondence with tau pathology (Enwright et al., 2022). SK3 channel expression increases with age in the mouse hippocampus, and mediates the reductions in LTP with age (Blank et al., 2003). Reduced neuronal firing due to excessive intracellular Ca^2+^ is also seen in aged rat hippocampus (29–31 mos) (Oh et al., 2013), and aged rat entorhinal cortex (Gant et al., 2018). Note that these animals were much older than mice typically used in AD models (<12–14mos) (Zhong et al., 2024). Thus, *hyper*excitability may predominate in mouse AD models as they are often studying genetic manipulations in a young brain. The high levels of SK3 channels in primate association cortex suggests that this loss of firing may occur even at younger ages in human association cortices.

An additional complication is that recordings of neurons *in vitro*, eg in slices or biopsy material, do not always reflect neuronal activity *in vivo* when the circuit is engaged in a cognitive task. For example, *in vitro* recordings from layer III dlPFC neurons from macaque show that they have increased firing rates, and also increased late afterhyperpolarization of the action potential (Chang et al., 2005; Luebke and Amatrudo, 2012), reflecting increased potassium channels opened by Ca^2+^ (e.g., SK, IK channels) and/or PKA (e.g., KCNQ2) (Sahu and Turner, 2021). However, *in vivo* these neurons show reduced firing with age when recorded from macaques performing a working memory task, and firing can be improved by blocking potassium channels (Wang et al., 2011). Thus, claims of hyperexcitability in AD must be viewed with caution if using *in vitro* recordings, or if from circuits in mouse models with lower expression of SK potassium channels than in humans.

Recent proteomic/transcriptomic studies have posited that there is hyperexcitability in AD due to a loss of some interneurons early in AD: specifically SST-expressing interneurons and layer I reelin/NDNF expressing (Gabitto et al., 2024; Gazestani et al., 2023). However, this is a relatively small subset of interneurons, and the reduced activity of numerous afflicted pyramidal cells may override and produce a generalized hypoactive state.

Altogether, this is a complex arena where assumptions about *in vitro* circuit activity should be made with caution given differences in methods, circuits, species and the relative expression of SK channels that can make intracellular Ca^2+^ reduce, rather than increase, neuronal firing.

## Interactions between intracellular Ca^2+^ dysregulation and pTau pathology

As described above, intracellular Ca^2+^ dysregulation can increase tau pathology through multiple mechanisms. It can increase tau hyperphosphorylation through direct activation of PKC (Ekinci and Shea, 1999), and CamKII (Wang et al., 2007a), through indirect activation of PKA, and by cleavage and disinhibition of GSK3β (Jin et al., 2015) and of p35 to p25 which activates cdk5 as well as GSK3β (Chow et al., 2014; Lee et al., 2000). The ratio of p25/p35 increases early in the course of AD (Kurbatskaya et al., 2016), consistent with elevated calpain activity being an early driver of pathology. Calpain and caspases also truncate tau itself which renders it more vulnerable to post-translational modifications (Rao et al., 2014).

Tau phosphorylated at pT217Tau is of special interest as it is an emerging plasma biomarker that is evident very early and indicates that there is soluble pTau arising in the human brain at earlier stages than previously expected (Barthélemy et al., 2024; Palmqvist et al., 2020). ImmunoEM of the aging macaque shows aggregations of soluble pT217Tau accumulating in spines and on the SER and microtubules of dendrites in the early aged ERC and the late aged dlPFC. Aggregations on microtubules interfere with endosomal trafficking which may weaken dendritic integrity (Datta et al., 2024a) (Figures 4c, 6d). Consistent with this, pT217Tau is often in dendrites with large numbers of autophagic vacuoles (Datta et al., 2024a) (Figures 4c, 6e). With very high magnification, one can see pT217Tau “seeding” between neurons, where it is exposed to the extracellular space for capture in CSF and plasma (Datta et al., 2024a) (Figure 6f). These data suggest that even early stage, soluble pTau is harmful to neurons.

In addition to the extensive evidence that excessive intracellular Ca^2+^ increases tau pathology, there is some evidence that pTau increases Ca^2+^ dysregulation, thus driving vicious cycles. For example, abnormal tau is associated with increased Ca^2+^ in motor neurons that can be partially normalized by removing pathological tau (Wu et al., 2021). Similar effects have been seen in the giant squid axon (Moreno et al., 2016). Application of tau aggregates *in vitro* increases Ca^2+^ entry through voltage-gated Ca^2+^ channels and causes reactive oxygen species and neuronal death (Esteras et al., 2021).

## Interactions between intracellular Ca^2+^ dysregulation and Aβ_42_ pathology

As described above, high levels of intracellular Ca^2+^ signaling can increase Aβ_42_ generation through calpain cleavage of p25-cdk5 signaling, which increases the activity of β-secretase (Wen et al., 2008). Human-induced neurons from AD patients with PS1 mutations had increased Aβ_42_ expression that was reduced by blocking Ca^2+^ release from the SER, suggesting that internal Ca^2+^ release plays a role in amyloid genesis in autosomal dominant PS1 AD (Schrank et al., 2020).

There is extensive evidence that Aβ_42_ can increase Ca^2+^ dysregulation, which may indeed be a large part of how Aβ_42_ increases tau hyperphosphorylation and other toxic actions. For example, Aβ_42_ oligomers can form artificial ion pores that flux Ca^2+^ into the cell (Small et al., 2009). Aβ_42_ also increases Ca^2+^ release from the SER (Demuro et al., 2005; Demuro and Parker, 2013; Demuro et al., 2010). There is also evidence that soluble Aβ_42_ increases Ca^2+^ entry through NMDAR (Arbel-Ornath et al., 2017), and that it induces Ca^2+^ overload of mitochondria (Calvo-Rodriguez et al., 2020; Naia et al., 2023). In addition to multiple actions by Aβ_42_ itself, the APP intracellular domain liberated upon the cleavage of Aβ_42_ from APP also increases Ca^2+^ release from the SER (Hermes et al., 2010). These multiple mechanisms to increase cytosolic Ca^2+^ can then engage the actions to cause tau pathology (described above) and autophagic degeneration (described below).

## Interactions between pTau and Aβ_42_ pathology

It is well substantiated that Aβ_42_ can increase tau phosphorylation, and indeed this is a key tenet of the Amyloid Hypothesis (Selkoe and Hardy, 2016). However, our data from aged macaques indicate that the converse is also true, and that aggregations of soluble pTau on microtubules may increase the production of Aβ_42_ (reviewed in Arnsten et al., 2021a; Arnsten et al., 2025; Datta and Arnsten, 2025). This hypothesis is based on parallel studies of which show that insults that slow endosomal trafficking lead to increased amyloid pathology. For example, genetic impairments in *SORL1* are a risk factor for AD and these lead to slowed endosomal trafficking (Knupp et al., 2020). Previous studies have shown that APP is more likely to be cleaved to Aβ_42_ in endosomes, which express β-secretase (Cataldo et al., 1997). Thus, slowing endosomal trafficking leads to more time with APP exposed to β-secretase and thus the production of Aβ_42_ (Bhalla et al., 2012; Nixon, 2005; Small et al., 2017).

Our data from aging macaques indicates that aggregations of soluble pTau on microtubules also may slow endosomal trafficking and thus contribute to greater Aβ_42_ production. Nanoscale immunoEM imaging shows soluble pTau surrounding endosomes that contain APP, likely slowing their progress and “trapping” APP near β-secretase (Paspalas et al., 2018). For example, one can see Aβ_42_ in enlarged endosomes that are surrounded by pT217Tau in dendrites (Datta et al., 2024a).

We have hypothesized that a prolonged period of soluble pTau may be needed to generate very high levels of Aβ_42_, while conditions that cause rapid fibrillation of tau and destruction of the dendrite would destroy the engine needed to generate amyloid pathology (Arnsten et al., 2021a; Arnsten et al., 2025; Datta and Arnsten, 2025). Thus, conditions such as Frontotemporal Dementia may have little or no amyloid pathology as the tau fibrillation occurs so rapidly, destroying the engine for Aβ_42_ production. A similar explanation may clarify why there is relatively little amyloid pathology in the ERC in AD, as tau pathology and autophagic degeneration proceed so quickly in this cortical region. Conversely, there is a prolonged period of soluble pTau in the aging association cortices, which may contribute to the extensive Aβ_42_ in these regions.

## The role of intracellular Ca^2+^ dysregulation in autophagic degeneration

Lysosomal activity and autophagy have a complex role in neurodegenerative disorders, where their healthy operations are needed to breakdown debris and provide nutrients, but excessive, or misplaced activity can slowly destroy neurons (Ferguson, 2019; Nixon and Rubinsztein, 2024). Data from rodent models suggest that basal levels of internal Ca^2+^ release via RyR2 are needed for healthy autophagy, e.g., clearing amyloid accumulation (Zhang et al., 2023). However, excessive Ca^2+^ leak and cytosolic calcium levels sufficient to activate calpain-2 may increase autophagic degeneration (Nixon and Rubinsztein, 2024; Vervliet, 2018). Calpain-2 cleaves and activates heatshock protein 70.1 (hsp70.1) which initiates multiple actions that degrade the neuron (schematically shown in Figure 4b). Hsp70.1 increases autophagic degeneration, which is the process by which neurons degenerate in AD (Okamoto et al., 1991), and it also permeabilizes the lysosomal membrane, causing a loss of acidification and thus a loss of lysosomal function and the release of lysosomal cathepsins into the cytoplasm (Sahara and Yamashima, 2010; Yamashima, 2013; Yamashima et al., 2024). This has been documented in neurons from AD patients (Nixon et al., 2005), as well as enlarged endosomes (Cataldo et al., 1997) consistent with pTau interfering with endosomal trafficking.

## APOE-ε4 genotype exacerbates many aspects of intracellular Ca^2+^ dysregulation and inflammatory signaling

APOE is a key protein in the brain, responsible for lipid and cholesterol metabolism (Holtzman et al., 2012; Raulin et al., 2022). Its three main alleles—ε2, ε3, and ε4—carry varying disease risks, with APOE-ε4 being the strongest genetic risk factor for sAD, increasing risk by up to 15-fold in homozygotes (Corder et al., 1994; Martens et al., 2022; Strittmatter et al., 1993; Zalocusky et al., 2021). APOE-ε4 is associated with higher levels of Aβ_42_ and tau pathology, neurodegeneration, and with increased Ca^2+^ dysregulation, while APOE-ε2 offers protection against dementia and reduces AD risk (Morrison et al., 2024; Serrano-Pozo et al., 2015). APOE-ε4 promotes more Aβ plaques, earlier onset of amyloid pathology, and more widespread cortical amyloid aggregation, compared to ε2 and ε3 (Gonneaud et al., 2016; Liu et al., 2017; Mishra et al., 2018; Murphy et al., 2013). APOE-ε4 also increases greater cytotoxic Aβ42 aggregation and oligomerization (Garai et al., 2014), which may be targeted in immunotherapy for early-stage sAD (Huynh et al., 2017). APOE-ε4 also exacerbates microglial actions that propel atrophy (Rosenzweig et al., 2024; Shi et al., 2019; Yin et al., 2023). APOE-ε4 also increases tau pathology (Thierry et al., 2024), e.g., exacerbating tau phosphorylation (Shi et al., 2017; Wadhwani et al., 2019). APOE-ε4 is associated with increased tau pathology in brain (Young et al., 2023), and in fluid biomarkers, i.e., CSF (Benson et al., 2022), and pT217Tau levels in blood (Pandey et al., 2025). APOE-ε4 also hastens tau pathology in another tauopathy, FrontoTemporal Dementia (Koriath et al., 2019). In contrast, the protective “Christchurch” mutation of APOE-ε3 reduces tau pathology (Chen et al., 2025; Sepulveda-Falla et al., 2022).

Many of the detrimental effects of APOE-ε4 likely involve its aggravation of Ca^2+^ dysregulation, e.g., caused by inflammatory processes (Wang et al., 2022). For example, APOE dose-dependently increases free intraneuronal Ca^2+^ levels in the order of: APOE-ε4 > APOE-ε3 > APOE-ε2, in line with their risk for increasing sAD (Ohm et al., 2001). APOE-ε4 causes a sustained increase in intracellular Ca^2+^ levels by activating both NMDARs and L-type voltage-gated Ca^2+^ channels (L-VGCCs) (Ohkubo et al., 2001; Ramakrishna et al., 2021). APOE-ε4 also increases Ca^2+^ release from the smooth endoplasmic reticulum (SER) via ryanodine receptors (Ohkubo et al., 2001). APOE-ε4 also impairs Ca^2+^ handling in lysosomes contributing to degeneration (Larramona-Arcas et al., 2020). *In vitro* studies have shown that the intracellular increase in Ca^2+^ caused by APOE-ε4 is associated with increased tau pathology (Wadhwani et al., 2019) and increased cell death (Jiang et al., 2015; Veinbergs et al., 2002). Consistent with these findings, APOE-ε4 has been shown to increase calpain, p35/p25 and cdk5 expression (Zhou et al., 2016), which would increase tau, amyloid and autophagic pathology (Figures 4, 5).

## Restoring regulation of intracellular Ca^2+^signaling as a therapeutic strategy

**Figure 7:**
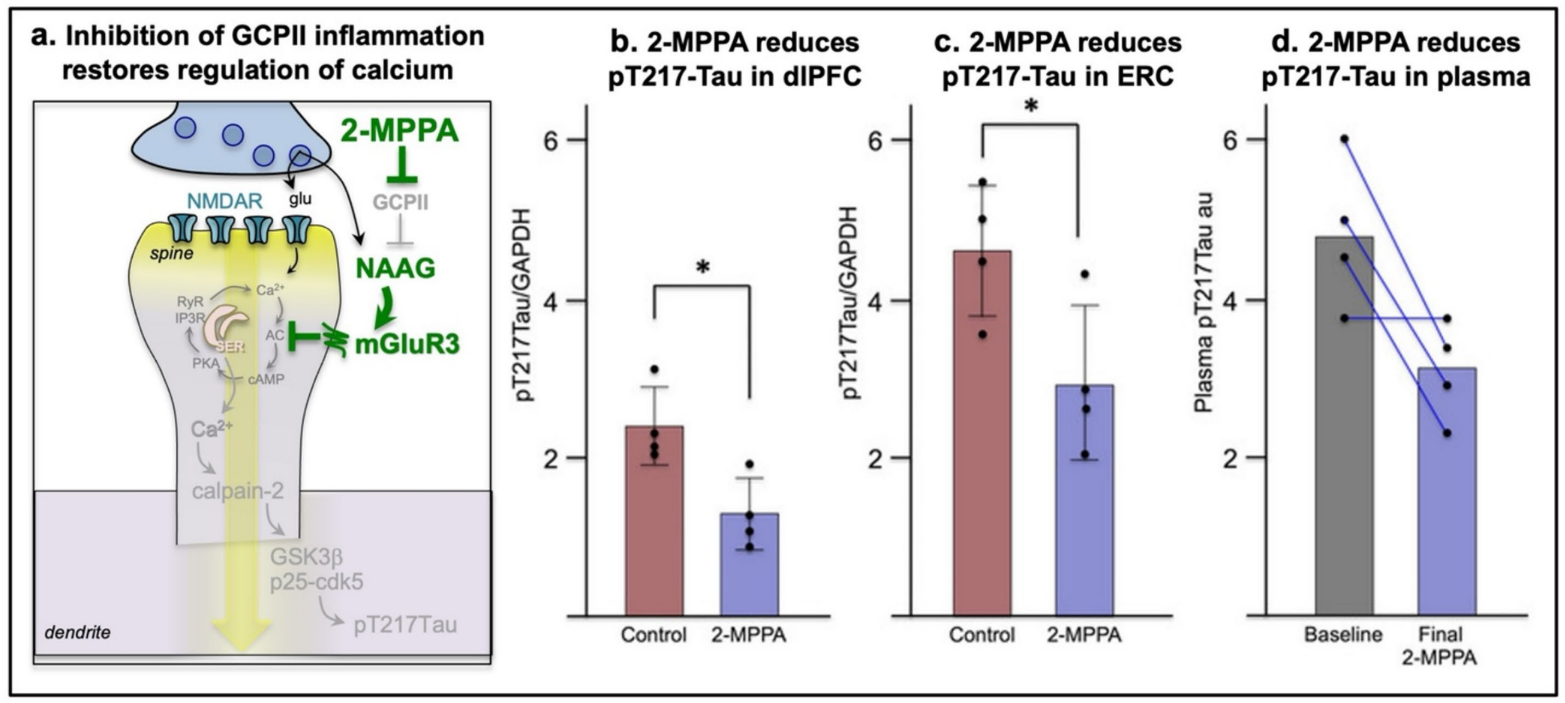
Chronic inhibition of GCPII inflammation reduces pT217Tau in aged macaques. **(a)** Schematic diagram showing how the GCPII inhibitor, 2-MPPA, restores mGluR3 regulation of cAMP-Ca^2+^ signaling and reduces tau hyperphosphorylation. **(b)** Six months daily with 2-MPPA reduces pT217Tau levels in the dlPFC of aged macaques. **(c)** Six months daily treatment with 2-MPPA reduces pT217Tau levels in the ERC of aged macaques. **(d)** Six months daily treatment with 2-MPPA reduces pT217Tau levels in the plasma of aged macaques compared to baseline levels. From Bathla et al. (2023).

Given the destructive effects of excessive intracellular Ca^2+^ signaling, restoring the regulation of Ca^2+^ may be a helpful strategy to reduce the toxic effects of inflammation on higher cortical circuits and reduce the risk of sporadic AD (reviewed in Datta and Arnsten, 2025). As inflammation appears to be a major risk factor for sporadic AD, agents that inhibit the inflammatory pathways that drive Ca^2+^ dysregulation may be a particularly helpful strategy for protecting the aging brain. Although there has been little focus on postsynaptic mGluR3 regulation of Ca^2+^ signaling, perhaps due to its more limited role in rodents (Woo et al., 2022), GCPII inflammatory erosion of mGluR3 signaling appears to be a large contributor to cognitive impairment in both nonhuman and human primates (Wiseman et al., 2025; Yang et al., 2022; Zink et al., 2020). As described above, GCPII activity in the aged macaque dlPFC highly correlates with levels of pT217Tau (Figure 5b). Recent data show that inhibition of GCPII in macaques greatly enhances dlPFC neuronal firing and spatial working memory abilities (Yang et al., 2022), and chronic daily administration of a GCPII inhibitor for 6 months significantly reduces pT217Tau levels in dlPFC and ERC and in plasma (Bathla et al., 2023) (Figure 7). There was no evidence of side effects, consistent with a potential therapeutic (Bathla et al., 2023). Thus, restoring mGluR3 regulation of Ca^2+^, or other mechanisms that can reduce the toxic effects of excess calcium, may be especially helpful in protecting the aging brain from AD pathology. Additional strategies to reduce toxic Ca^2+^ actions could include direct inhibition of calpain-2 (Arnsten and Baudry, 2023), or indirect inhibition of calpain-2 by stimulating the p75 neurotrophin receptor (McCollum and Estus, 2004), a treatment strategy which shows early promise (Yang et al., 2020).

## Summary- feedforward interactions between Aβ_42_, pTau and Ca^2+^/inflammation

**Figure 8:**
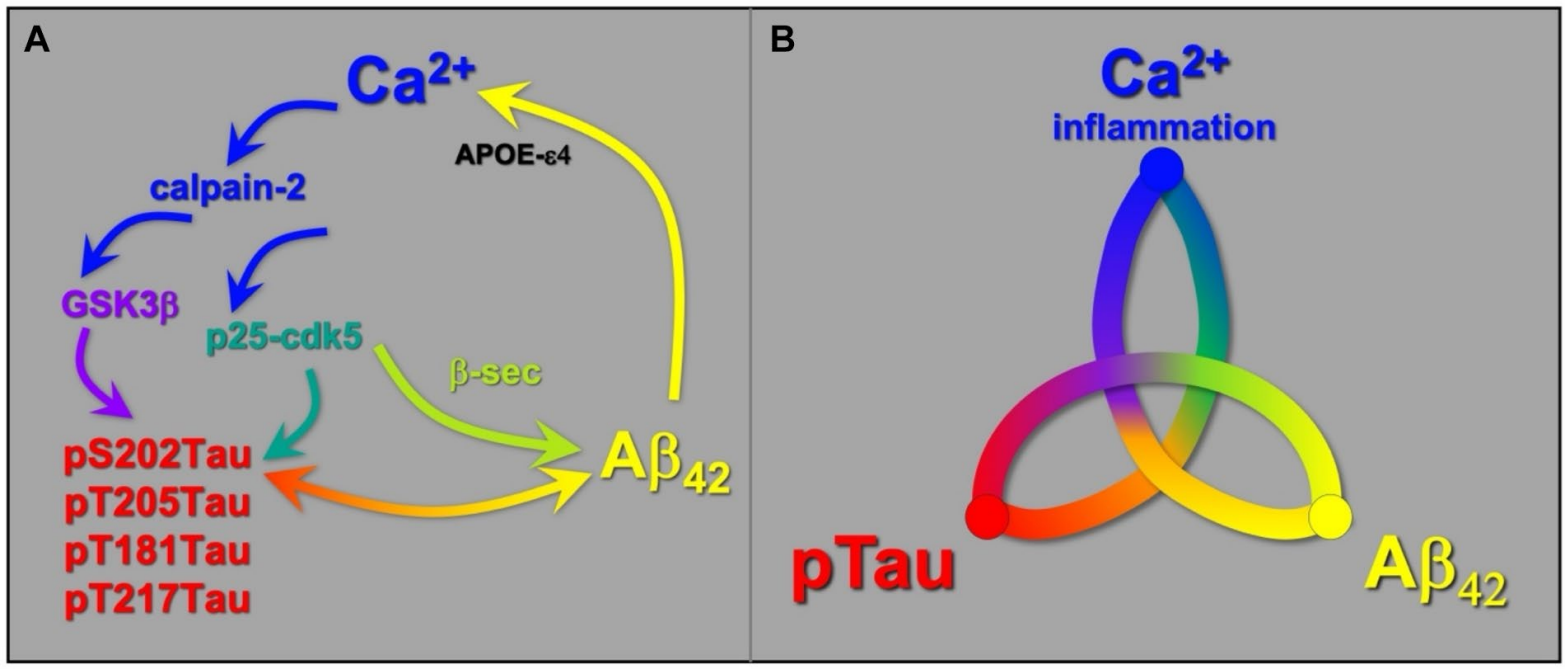
Schematic diagram showing that Ca^2+^ dysregulation, Aβ_42_ and pTau pathology all exacerbate each other, creating feedforward signaling that propels pathology. **(A)** Examples of signaling events that drive feedforward pathological actions (see text for further details). **(B)** A schematic showing the three-way interactions between Ca^2+^ (inflammation), pTau and Aβ_42_ pathologies.

In summary, a detailed analysis of the aging primate association cortex shows that the processes of sporadic AD pathology are nonlinear and interactive, with dysregulated intracellular Ca^2+^ increasing tau and amyloid pathologies, tau and amyloid increasing Ca^2+^ dysregulation, and tau and amyloid pathologies each worsening the other (Figure 8). This differs from the Amyloid Hypothesis, which posits that Aβ_42_ is the sole initiating event. Although Aβ_42_ increases Ca^2+^ dysregulation to mediate many of its toxic actions (Demuro et al., 2010), it is also clear that Ca^2+^ dysregulation can arise from other sources, e.g., inflammation and stress, which may be particularly important factors in sporadic AD. Thus, initiating factors in sporadic AD could arise from multiple origins that all engage feedforward signaling events, ultimately leading to a similar common phenotype.

Given the multiple, feedforward signaling events, early intervention may be key to prevention of disease prior to the loss of neuronal integrity. Restoring regulation of Ca^2+^ signaling may be particularly helpful given how central Ca^2+^‘s toxic actions are to multiple AD pathologies. The advent of an early marker of ensuing AD, plasma pT217Tau, may now provide a feasible strategy for testing potential preventive strategies prior to significant neuronal damage.

In closing, we see that the very molecular events needed for higher cognition and memory formation render neurons especially vulnerable to AD pathology when Ca^2+^ is dysregulated by age and inflammation. It is hoped that this knowledge can now enable strategies to restore and protect our fragile higher circuits at the top of the cortical hierarchy.
